# Investigation of the Warpage of a High-Density Polyethylene Pallet by Plastic Injection Compression Molding: Part I—Numerical Approach

**DOI:** 10.3390/polym14071437

**Published:** 2022-04-01

**Authors:** Chun-Der Cheng, Yi-Ling Liao, Hsi-Hsun Tsai

**Affiliations:** 1 Department of Mechanical Engineering, Ming Chi University of Technology, New Taipei City 24301, Taiwan; cdcheng@mail.mcut.edu.tw (C.-D.C.); yiling@mail.mcut.edu.tw (Y.-L.L.); 2 Research Center for Intelligent Medical Devices, Ming Chi University of Technology, New Taipei City 24301, Taiwan

**Keywords:** warpage, plastic pallet, injection compression molding, flatness, molding flow analysis

## Abstract

Many challenges are associated with the injection compression molding process for producing a half-pallet (1320 mm × 1110 mm × 75 mm, length × width × height), which is butt-welded to another one for enhancing its strength. This pooled high-density polyethylene (HDPE) pallet is able to endure the impacts of a heavy load and a low ambient temperature. Reducing the warpage of a half-pallet is, therefore, essential for reducing the residual internal stress within the welded portions. An advanced Moldex3D package helps to detail the temperature distribution and warpage of a half-pallet. The pre-setting molding parameters from a mass-production factory produce half-pallets with worse flatness. In this investigation on using appropriate cooling water temperatures within the core and cavity plates of the mold, the numerical results show that the warpage of the top surface of the half-pallet was 11.549 mm, low warpage with respect to this large-scale pallet. Furthermore, the compression speed of 50–60 mm/s may have produced a low flatness of the half-pallet in this study.

## 1. Introduction

A pallet, a base for assembling, loading, storing, handling, stacking, transporting, or displaying goods and loads [1], has three categories: single-use, buy/sell, and pooled [2]. Only the pooled pallet has a reinforced structure for loading in heavy-duty conditions in a warehouse with forklifts and usually has a specific color for identification throughout the supply chain. The life cycle and the carrying load of the trans-shipment are the typical concerns of a pooled pallet. For trans-shipment, polypropylene (PP) and polyethylene (PE) are usually applied in plastic pallets due to their good mechanical performance. In the cryogenic environment of a warehouse, the PE pallet is suitable for carrying goods since the glass transition temperature of PE is lower than that of PP. PE has good performance in terms of cryogenic impact strength [3]. Because of its high molecular weight, HDPE shows high performance in terms of mechanical properties [4]. However, because of the high molecular weight, it requires a high processing pressure due to its high melt viscosity and lack of fluidity [5]. Therefore, occasionally, injection compression molding (ICM) is used instead of injection molding for an HDPE pooled pallet. Combining the techniques of injection and compression molding, the mold is not closed completely at the filling stage and starts to fully close once the melt polymer has partially filled the cavity. Because of its high melt viscosity, the sizes of the rib features in the HDPE pallet by ICM are greater than that of the one by injection molding, which addresses its requirements regarding heavy-duty endurance.

By differential scanning calorimetry (DSC) and X-ray diffraction (XRD), a tensile specimen of HDPE formed by ICM was found to have a higher yield strength and Young’s modulus than the one formed by injection molding. These mechanical properties of the specimen are proportional to its crystallization percentage [6]. The tensile properties were significantly reduced with an increasing temperature, while the elastic modulus and the ultimate tensile strength linearly increased at higher strain rates. In addition to the elastic modulus, yield, and ultimate strengths, the polynomial relations for the tensile properties were developed as functions of the strain rate and temperature. These relationships could be used to estimate the tensile strength of HDPE as a function of the temperature and strain rate in the design phase [7]. In ICM, polymer flow behaviors under compression, such as the power-law index and curve-fitting rheological parameters, are almost constant in comparison to the numerical results from Moldex3D and the experimental relationship between the loading force and the displacement [8]. The aforementioned studies have indicated that the mechanical properties of HDPE are positively proportional to the crystallization rate and functions of the temperature and strain rate. The power law and rheological parameters of polymer flow behavior under compression remain constant. For application in a warehouse with forklifts, the pooled pallet has two loading sides that are horizontally symmetrical and four ways of entry for the forklift. The pooled pallet has a horizontally symmetrical shape, so a half-pallet is designed to reduce the manufacturing cost. Bonding two half-pallet pieces derived from ICM by heating using a hot plate [9] into a whole pooled pallet by plastic welding may create a strong structure to address the requirements of a pooled pallet. The surfaces of the joined portions of each half-pallet are heated via direct contact with a hot metallic plate. Once the temperatures of the joined portions reach the pre-set threshold value, the acting pressure on each side of the half-pallet leads to welding through molecular chain diffusion and the formation of molecular entanglements at the interface of the joint region [10]. However, the interface of the joined portion between the two half-pallets might have a large amount of internal stress if each half-pallet warps after the demolding stage of ICM. Warpage is thus vital for enhancing the quality of the injection part that otherwise creates problems that are subject to poor material characterization and inadequate control parameters [11,12,13,14].

The cooling rate of the molded parts within the mold cavity may affect the relaxation and reorganization levels, leading to an asymmetric distribution of mechanical properties [15]. Plastic injection molding involves four major stages—filling, packing, cooling, and ejection—while ICM involves filling, compressing, cooling, and ejection. The compression rate, the cooling channel temperature, the cooling time, and mold temperatures may affect the warpage of the molding parts, where the mold temperature is the main factor [16]. The mold temperature is affected by the cooling channel temperature, the cooling time, and the demolding time. After demolding, the parts ejected from the core plate are free to deform and are cooled down to ambient temperature by the heat convection effect. Internal stresses occur in the molded parts once there are different temperature gradients around the parts. These different temperature gradients may cause internal stress and warpage. Because the temperature in some parts is still high, these parts are not stiff and immediately warp toward the cold side upon ejection. The hot side of the pallet has a higher temperature gradient than the cold side; it is less stiff [17]. Simulations of a disk part in ICM showed that a faster compression speed, a smaller compression stroke, a smaller switch time, and a thinner part would result in shrinkage of the lower part. In addition, the same compression pressure in the post-filling stage would result in improved shrinkage reduction due to the melt-temperature effect introduced at the end of the filling stage [18]. By ICM, the warpages of two types of rectangular plates with orthogonally stiffened ribs of sizes 1800 mm × 600 mm × 12 mm and 1200 mm × 600 mm × 12 mm were simulated to compare with the experimental results. They revealed that the warpage of the molded part is inevitable due to the thermal shrinkage of polymeric materials and the large size of the product, and all the numerical predictions were in good agreement with the experimental results. However, the study did not provide quantitative results [19]. A numerical approach was used for three-dimensional flow during the compression stage in ICM. Compression gap and velocity play significant roles in the pressure on the filling gate for lens molding [20]. The flow-induced stress during the compression stage was also simulated to find that the flow-induced stress is proportional to the compression velocity and a higher melting temperature decreases flow-induced stress [21].

Either in the injection molding process or in ICM, multiple physical effects on the molding parts due to operational parameters were analyzed using the following commercialized software: ANSYS Fluent [22,23], Moldflow [24], Open FOAM [25], and Moldex3D [13,26,27,28,29,30]. Numerical investigation was implemented using Moldex3D software. Both the skin and core materials are considered in this mold flow software to be compressible, generalized non-Newtonian fluid. A modified-cross model with Arrhenius temperature dependence is used to describe the viscosity of the polymer melting materials. The finite volume method for a discrete Navier–Stokes equation was used to solve the transient flow field in a three-dimensional coordinate in Moldex3D [31]. The three linearized momentum equations were then solved based on a guessed pressure field, followed by a solution of the pressure correction equation until a convergent result was derived [32].

Previous studies have blamed the traditional injection molding issues on the PP pallets of the single-use type and the buy/sell type. By ICM, the rectangular plates with orthogonally stiffened ribs of sizes 1800 mm × 600 mm × 12 mm and 1200 mm × 600 mm × 12 mm have been investigated, but there has been no quantitative comparison. In addition, no investigation of the HDPE pallet fabricated by ICM has been found. HDPE has a lower thermal conductivity coefficient. Therefore, it needs a longer cooling time. Therefore, this study aimed to perform a numerical study of a pooled HDPE half-pallet by ICM for loading in heavy-duty conditions in a warehouse. Two half-pallet pieces were bonded by butt welding. The warpage of a half-pallet should be as low as possible to ensure less internal residual stress later within the bonding portion. Based on the HDPE rheological properties, each parameter of ICM was examined using the Moldex3D advanced package to identify its effect on the warpage of the half-pallet. Then, the optimal molding parameters for reducing the warpage were derived for the minimum pallet flatness.

## 2. Materials and Methods

**Figure 1:**
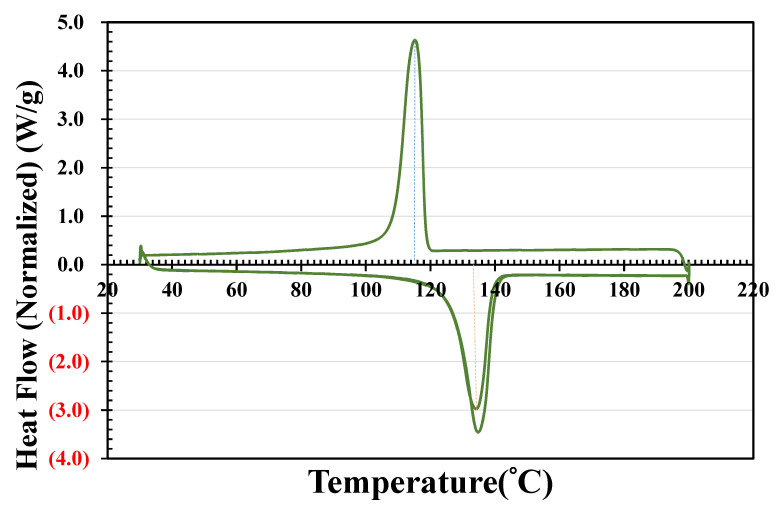
DSC heating curves of TAISOX^®^ HDPE 8041.

TAISOX^®^ HDPE 8041 [33] was used as the ICM material. The properties of TAISOX^®^ HDPE 8041 are as follows: density of 960 kg/m^3^, melt index (M.I.) of 4.0 under g/10 min, melting point of 133 °C, yield strength of 30.38 MPa, and Izod impact strength of 0.8 kg-mm/mm^2^ (i.e., 0.0784 J/mm^2^), followed by ASTM D638. In this study, the melting and crystallization behaviors of TAISOX^®^ HDPE 8041 were measured using a differential scanning calorimetry (DSC) instrument (TA Instruments Discovery DSC 25, New Castle, DE, USA) under a nitrogen atmosphere. TAISOX^®^ HDPE was heated to 200 °C under a ramp of 10 °C/min, held isothermal for 1 min, cooled to 30 °C at a rate of 10 °C/min, held isothermal for 1 min, and heated to 200 °C again at 10 °C/min for the crystallization and melting temperature measurements. In Figure 1, the TAISOX^®^ HDPE pellets analyzed by DSC depict starting melt temperatures of about 120 °C, a melting temperature range of 120 to 140 °C, and a crystallization temperature of 115.2 °C. The cooling enthalpy was 201.51 J/g and the second heating enthalpy was 199.61 J/g. The Izod impact strength of TAISOX^®^ HDPE 8041 was derived experimentally and was 1.35 kg-mm/mm^2^ (i.e., 0.1323 J/mm^2^), which is higher than the one from the previous data sheet. The flexural strength was also identified to be 2.49 kg/mm^2^ (i.e., 24.4 MPa).

**Figure 2:**
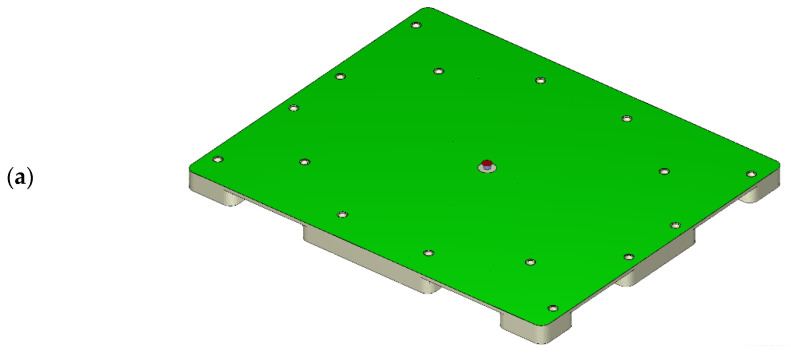

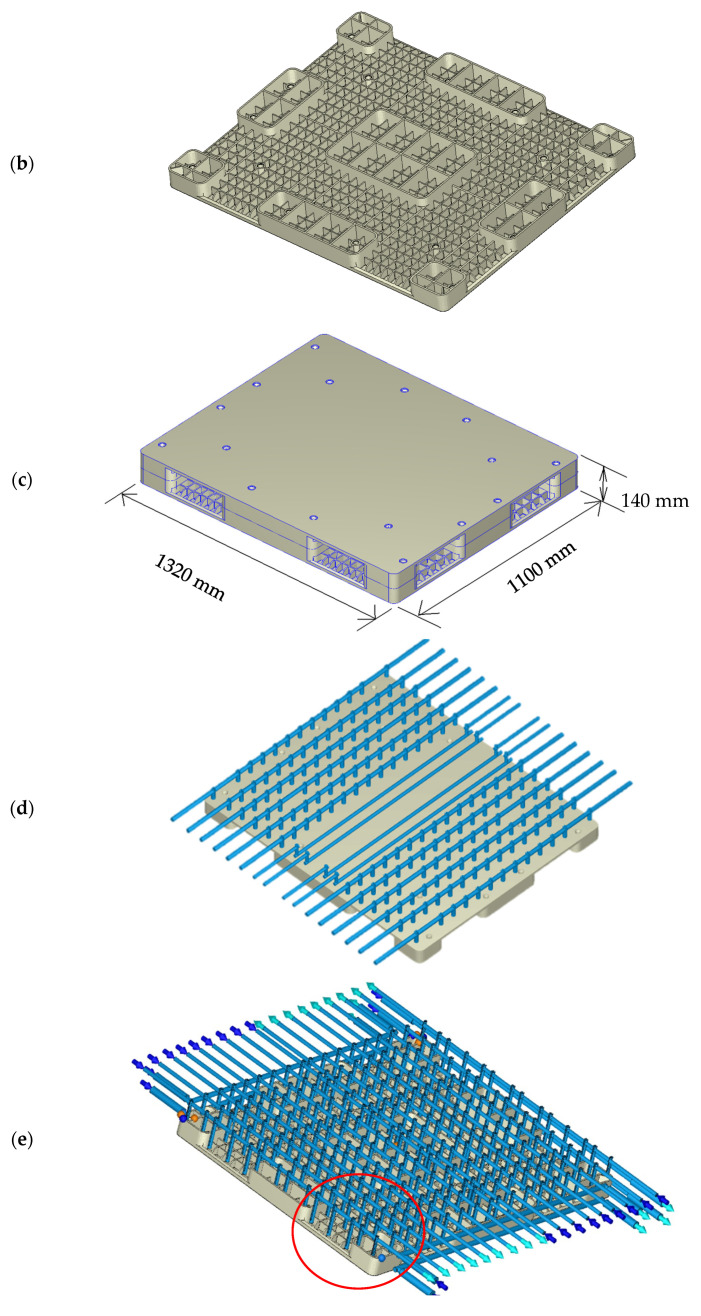

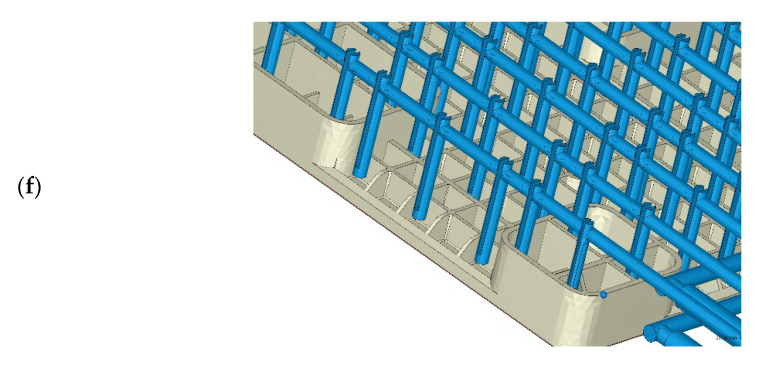
An injection-compression-molded HDPE half-pallet: (**a**) isometric view of the filling gate and the top side of a half-pallet (1320 mm × 1100 mm × 75 mm); (**b**) isometric view of stiffened ribs on the bottom side of a half-pallet; (**c**) dimensions of a welded whole HDPE pallet (1320 mm × 1100 mm × 140 mm); (**d**) isometric view of the baffle cooling flow system on the top side of a half-pallet; (**e**) isometric view of the baffle cooling flow system on the bottom side of a half-pallet; (**f**) detailed view of the baffle cooling flow system.

Figure 2 shows the dimensions of the HDPE pallet and the used cooling system within the mold in this study. The half-pallet had a smooth top surface and a rib-reinforced structure. Each rib was 3 mm thick. The diameter of the filling gate was 20 mm. Figure 2a shows the top side of a half-pallet with a filling gate 20 mm in diameter. The half-pallet was 1320 mm in length, 1100 mm in width, and 75 mm in height. The bottom side of the half-pallet had several reinforced ribs and legs, as shown in Figure 2b. Figure 2c shows an isometric view of a welded pallet, where the height of the welded pallet is 140 mm. Therefore, the welding butt should be 10 mm in height so that the two pieces of half-pallet add up to 150 mm. Differing from the straight cooling pipes for the cavity plate [31], the baffle cooling flow system was used for both the core and cavity plates, as shown in Figure 2d,e. A detailed view of the baffle cooling water channels of the bottom corner of the half-pallet is shown in Figure 2f, which would enhance the heat convention effects thanks to the larger surface of the baffle pipes.

A filling gate 20 mm in diameter allowed the low-viscosity molten HDPE material to flow into the mold cavity. This half-pallet had a volume of 19,993 cm^3^. The solid mesh of the half-pallet had 1,861,046 elements. The volume and mesh of the cold runner were 9.89 cm^3^ and 1120 elements, respectively. The associated cooling geometry contained 788,618 elements. As shown in Table 1, the injection parameters were a mold temperature of 60 °C, an ejected molded component of 85 °C, a filling time of 16.257 s, a compression time of 6 s, a cooling time of 148 s, a mold opening time of 10 s, and a cycle time of injection molding of 193.8 s. The mold started compressing the molten HDPE until the end of filling. A maximum compression pressure of 49.51 MPa occurred at the first second in the compression stage. Then the compression pressure gradually decreased to 17.05 MPa at the end of the compression stage. Under a low injection pressure, an ICM machine developed by Remaplan Anlagenbau GmbH, Landshut, Germany, was employed in this study (Remaplan Anlagenbau GmbH was terminated on 16 August 2006). This molding machine contains a 90 kW shredder and a tank with a diameter of 1.2 m manufactured by EREMA [34], an extruder with a screw diameter of 120 mm and a heat capacity of 46.5 kW manufactured by EREMA, and a press machine with a maximum compression force of 1500 tons. A Moldex3D advanced package for ICM was used to analyze this HDPE half-pallet molding [35]. The core technologies of Moldex3D regarding the computation methods and schemes of the multi-physics of plastic injection molding are provided in Chang et al. [31].

## 3. Results and Discussion

### 3.1. Molten HDPE Spreading during Filling and Compression

**Figure 3:**
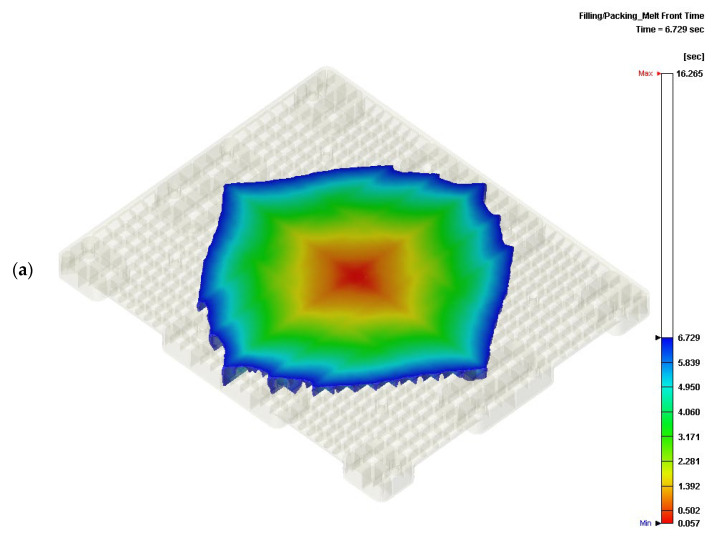

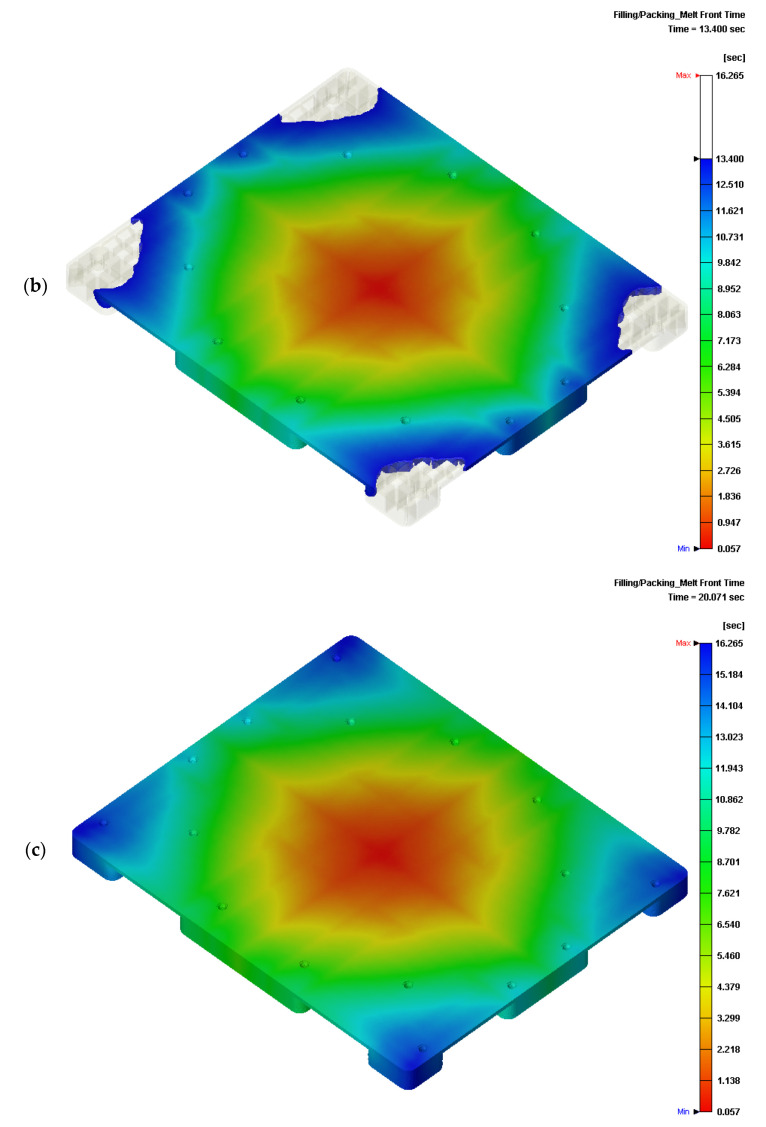
Spreading of the molten HDPE during filling and compression stages of ICM: (**a**) flow front from the filling gate at 6.729 s; (**b**) flow front from the filling gate at 13.40 s; (**c**) flow front from the filling gate at 16.265 s.

A total of 19,993 cm^3^ of the molten HDPE filled the cavity of the mold in 16.257 s. The average filling rate was 1.230 L/s. The compression speed of the core plate was 60 mm/s, where the compression gap was 1.0 mm. The flow front of the molten HDPE propagated to the corners of the cavity during the compression stage. The molten HDPE spread within 1.0 s under a maximum compression pressure of 49.51 MPa, as shown in Table 1. The activation of the filling gate was consistent, with 16.257 s needed for the molten HDPE to fill everything required. The flow front from the filling gate at 6.729 s is shown in Figure 3a. In the figure, the front is symmetric but not circular in pattern because reinforced ribs are needed under the surface of the pallet to fill with molten HDPE. At 13.40 s, in Figure 3b, the flow front penetrates into the cavity of the mold but the four corners are not yet filled. At 16.257 s, the compression took 0.1 s to fill the pallet, as shown in Figure 3c.

### 3.2. Pallet Profiles by Pre-Setting Molding Parameters

**Figure 4:**
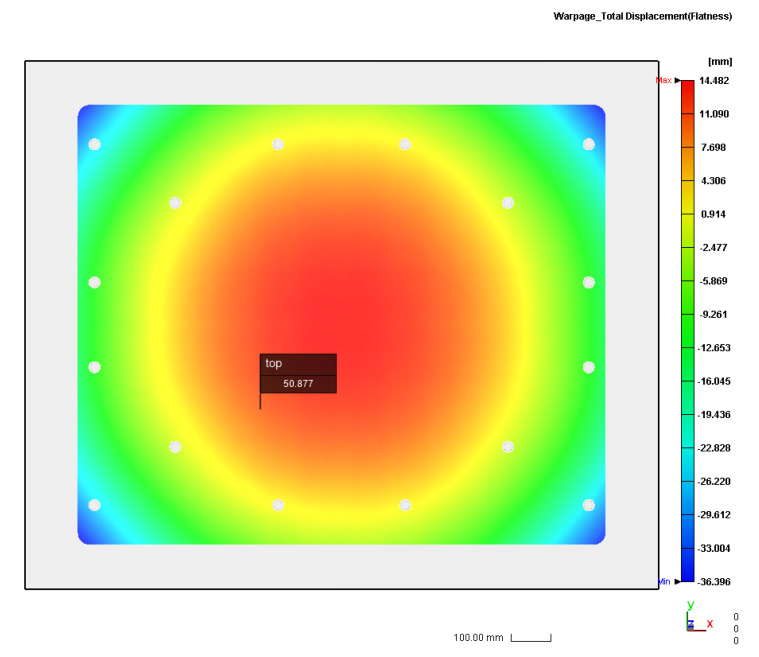
Top view of the numerically simulated total warpage of the HDPE half-pallet by ICM.

**Figure 5:**
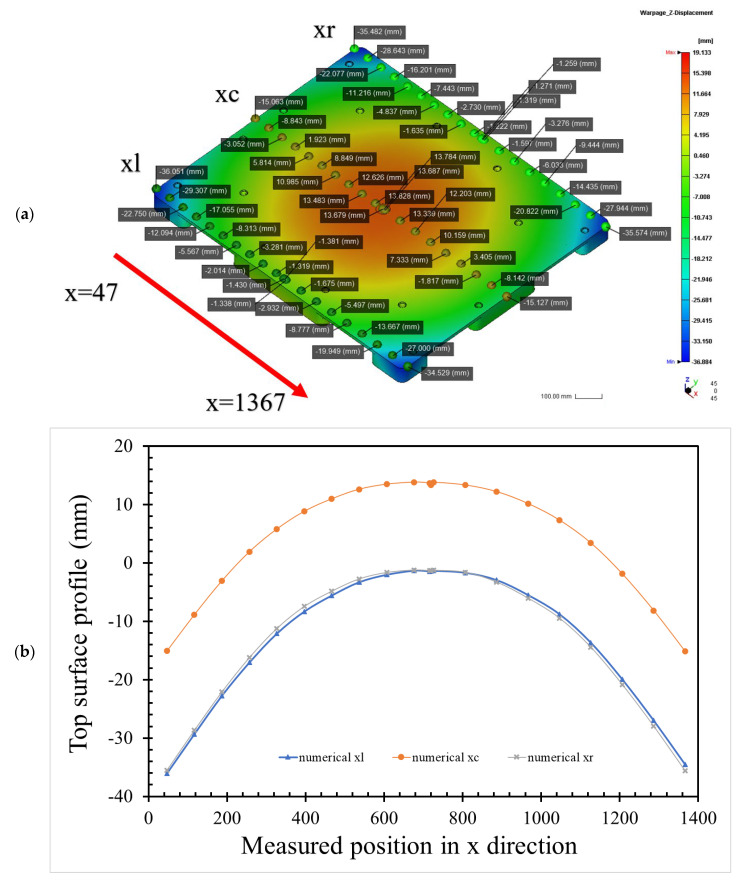

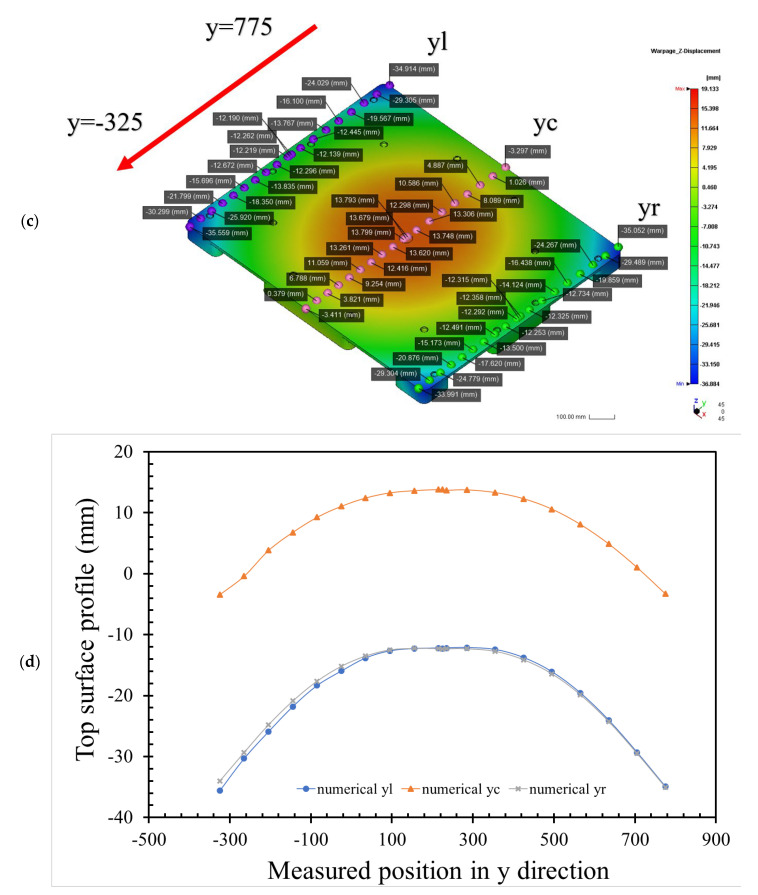
Top profiles in the height direction of the numerical warpage of an HDPE half-pallet by pre-setting ICM parameters: (**a**) isometric view of the numerical top flatness of the half-pallet along the x-axial direction; (**b**) x-axial top surface profiles; (**c**) isometric view of the numerical top flatness of the half-pallet along the y-axial direction; (**d**) y-axial top surface profiles.

According to the ICM parameters in Table 1, a numerical simulation of the half-pallet molding was performed using TAISOX^®^ HDPE 8041. The numerical simulation of the total warpage of the half-pallet is depicted in Figure 4, which depicts a convex profile and a flatness of 50.877 mm on the top surface of the pallet. This flatness of the half-pallet is high, so it is hard to load goods stably on it. A comparison of the numerical profiles of the injection compression-molded pallet along the *x*-axis is shown in Figure 5a, where the blue triangle denotes the simulated heights measured every 75 mm along the left-hand side (*x_l_*) of the top profile of the pallet. This profile is similar to the one on the right-hand side (*x_r_*) of the pallet, denoted by a gray cross. In Figure 5b, this similarity indicates that the half-pallet had symmetric warpage with respect to the filling gate. Either on the left-hand side or on the right-hand side, the deviation between the maximum and minimum heights of the profile was about 32 mm. However, the middle (*x_c_*) profile of the pallet had only a 28 mm deviation between the maximum and minimum heights, denoted by the orange dotted line.

Along the *y*-axis, the numerical heights of the profiles of the left-hand side (*y_l_*) and the right-hand side (*y_r_*) in Figure 5c also show a 24 mm deviation between the maximum and minimum heights of the numerical profiles, as shown in Figure 5d. The middle profile (*y_c_*) along the *y*-axis is numerically shown to be deformed by under 18 mm. The deviation in the y-axial direction is smaller than the one in the x-axial direction, thanks to a larger length of 1320 mm in the x-axial direction. These deviations in the profiles on the top surface of the pallet would not provide a stable base for carrying goods.

### 3.3. Proposed ICM Parameters for Lowering the Pallet’s Flatness

**Figure 6:**
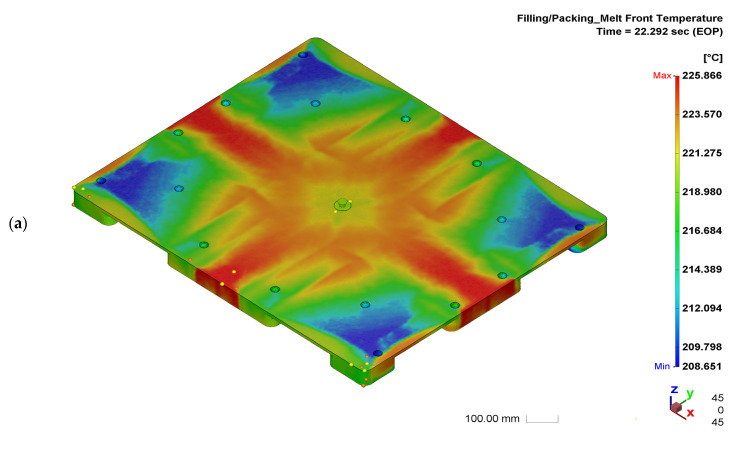

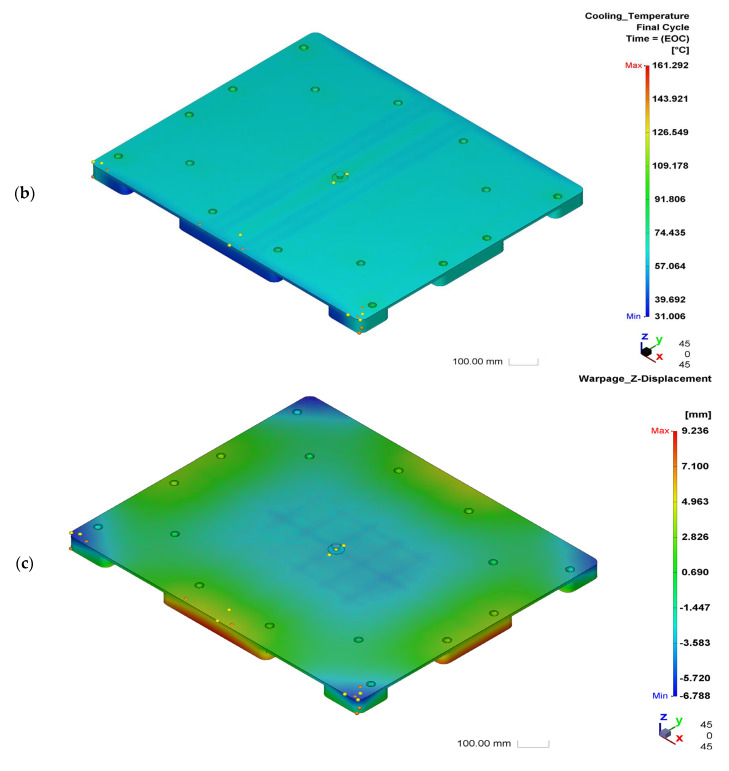

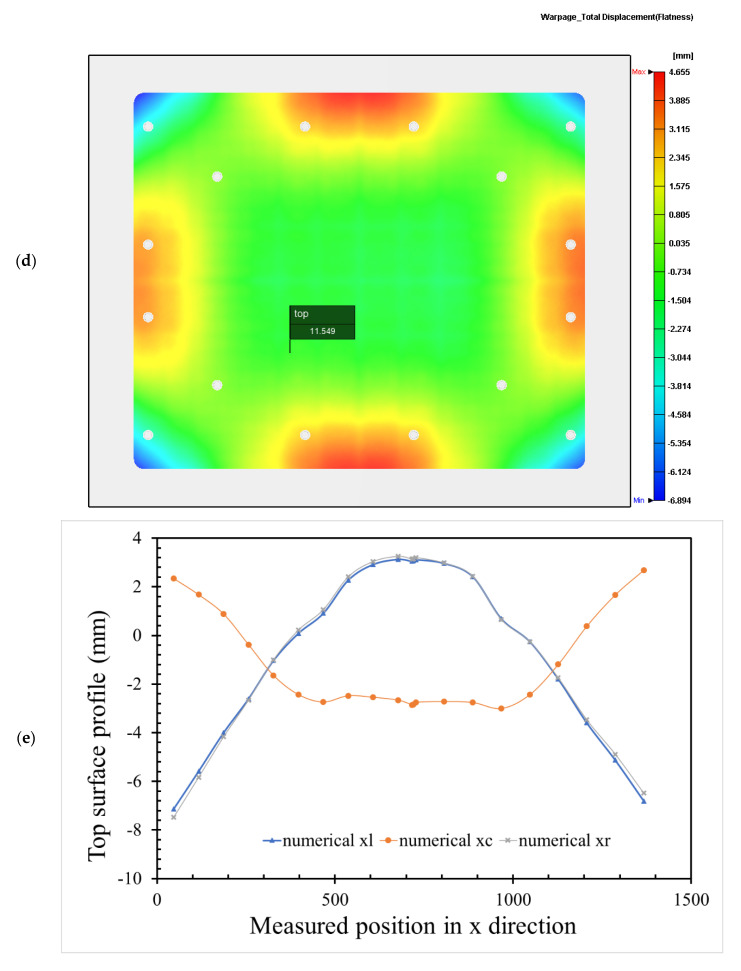

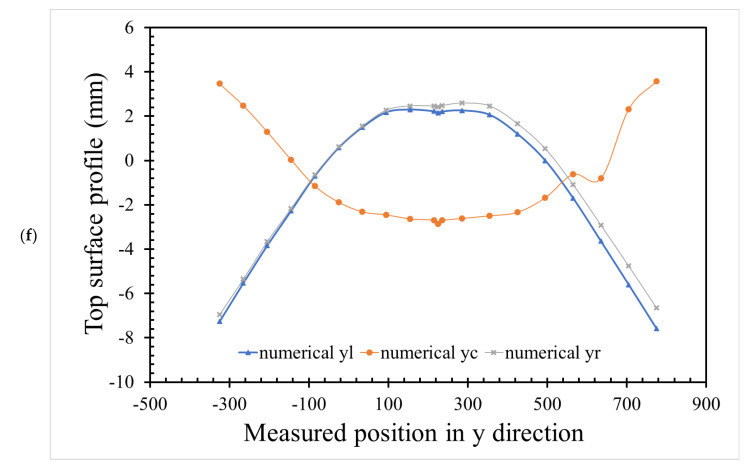
Simulated temperature distributions and warpage in the height direction of a half-pallet: (**a**) temperature distribution at the end of compression; (**b**) temperature distribution at the end of cooling; (**c**) isometric view of the numerical top warpage profile of the half-pallet; (**d**) flatness of the half-pallet top surface; (**e**) x-axial top surface profiles; (**f**) y-axial top surface profiles.

The pre-setting molding parameters induced a relatively high flatness of the half-pallet, making it difficult to maintain the stability of the stacked goods. An analysis of a simulation using different injection compression molding parameters that affect the flatness of the half-pallet was needed to achieve stability. The temperature distribution of the pallet under each stage of injection molding was acquired numerically by Moldex3D software. Under the same cooling water temperature on the cavity and the core plate of the mold in Table 1, the molded pallet had a numerical flatness of 50.877 mm, as shown in Figure 4. The molded HDPE pallet had no constraint to deform once demolded because the asymmetric internal stress occurring on the section of the pallet induced it to warp. For temperature gradients on the top and bottom surfaces of the pallet, the cooling water temperatures within the core and cavity plates should be different for smoothing the flatness. Since the hot side of the pallet had a higher temperature gradient after ejection from the mold around the room environment, a cooling-down effect by natural heat convection on the ejected pallet provided different temperature gradients on both sides of the pallet. The hot side may have deformed more than the cold side during the free quench of the pallet, leading to increasingly less warpage on the cold side. Therefore, the cooling water temperature within the cavity plate was 30 °C, and the one within the core plate was maintained at 60 °C. The temperature distribution of the HDPE half-pallet shows a non-uniform temperature at the moment of the end of the compression of ICM in Figure 6a. The deviation of the temperature on the top surface of the pallet was about 17 °C, where the maximum temperature was 225.866 °C. The shear strain rate of molten HDPE led to a temperature increase of 5.886 °C, due to which the molten HDPE was 220 °C before filling the mold.

The pallet temperature gradually decreased with respect to the cooling time. Before being ejected, the pallet was cooled continuously by heat conduction to the mold and heat convection to the cooling water. After a cooling period of 148 s, the temperature of the top surface of the pallet, as shown in Figure 6b, was nearly 60 °C and the average temperature of the cooled pallet was 66.3 °C. The numerical flatness of the molded half-pallet under these settings of cooling water temperature was only 11 mm in Figure 6c, which is much lower than that in the previous setting of 60 °C for all cooling water temperatures. This figure also shows a saddle shape of the warpage profile of the half-pallet. On the bottom side of the pallet, the flatness was about 11.549 mm, as shown in Figure 6c,d.

**Figure 7:**
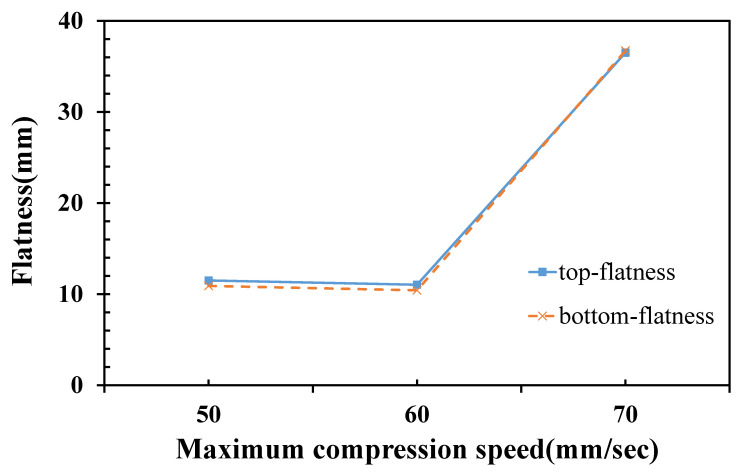
Numerical flatness with respect to the maximum compression speed for an HDPE half-pallet.

The warping trends of the numerical height deformation along the x- and y-axes are shown in Figure 6e,f. Simulated height deformations of the top half-pallet surface show that the outer part warped upward while the central part warped downward. Adjusting the temperature parameters may improve the flatness. The results of the simulated half-pallet are remarkably decreased, which can be used as a reference. According to the results of the analysis above, the ICM parameters would affect the flatness of the half-pallet. One of the differences between injection molding and ICM is the compression speed. By adjusting the compression speed and fixing the other ICM parameters, the flatness of the pallet shows a significant trend. The compression speed is, therefore, the focus of deriving a lower level of flatness because this parameter varies in a mass-production factory. Figure 7 shows the numerical flatness of the top surface of the HDPE half-pallet with respect to the compression speed during the compression of the ICM. The flatness of the top surface neared that of the bottom surface of the half-pallet. The flatness of the half-pallet was insensitive to a maximum compression below 60 mm/s, but it drastically increased from 11.028 to 36.517 mm above 60 mm/s at the maximum compression speed.

## 4. Conclusions

This study used mold flow analysis software to simulate the results of injection compression molding. By using different settings of the cooling water temperature within the core and cavity plates of the mold, the simulated warpage of the HDPE half-pallet decreased from 50.8 mm to 11.55 mm to ensure that the stacked goods are stable at a low ambient temperature. The warpage was relatively low and only characterized by a saddle shape of the half-pallet profile. Through simulation analysis, the compression speed of 50–60 mm/s provided a relatively low flatness of the half-pallet, but a compression speed of 70 mm/s may have enhanced the flatness. The greatest influence on the flatness of the half-pallet was setting different cooling water temperatures within the mold plates.

## Figures and Tables

**Table 1:** Injection compression molding parameters of TAISOX^®^ HDPE 8041.

Parameters	Conditions
Melt temperature (°C)	220
Mold temperature (°C)	60
Filling pressure (max) (MPa)	210
Filling time (s)	16.257
Filling/compression overlap (s)	1.007
Filling volume (mm^3^)	19,988,800
Compression speed (mm/s)	60
Compression gap (mm)	1
Compression time (s)	6
Max. compression pressure (MPa)	49.51
Cooling time (s)	148
Cooling channels’ temperature (°C)	30
Mold opening time (s)	10
Cycle time (s)	193.8

## Data Availability

All the data generated or analyzed during this study are included in the published article.
